# Transforming Growth Factor-β Concerning Malarial Infection and Severity: A Systematic Review and Meta-Analysis

**DOI:** 10.3390/tropicalmed7100299

**Published:** 2022-10-13

**Authors:** Kwuntida Uthaisar Kotepui, Pattamaporn Kwankaew, Frederick Ramirez Masangkay, Aongart Mahittikorn, Manas Kotepui

**Affiliations:** 1 Medical Technology, School of Allied Health Sciences, Walailak University, Tha Sala 80160, Thailand; 2 Research Excellence Center for Innovation and Health Product, Walailak University, Tha Sala 80161, Thailand; 3 Department of Medical Technology, Faculty of Pharmacy, University of Santo Tomas, Manila 1008, Philippines; 4 Department of Protozoology, Faculty of Tropical Medicine, Mahidol University, Bangkok 10400, Thailand

**Keywords:** transforming growth factor-β, TGF-β, severe malaria, uncomplicated malaria

## Abstract

Transforming growth factor-β (TGF-β) is important in the pathophysiology of malaria, but its role in acute and severe malaria is largely unknown. As a result, this study used a meta-analysis approach to investigate the difference in TGF-β levels between several groups of malaria patients and healthy controls. The systematic review protocol was registered at PROSPERO (ID: CRD42022318864). From inception to 7 March 2022, studies that reported TGF-β levels in patients with uncomplicated and healthy controls and patients with severe and uncomplicated malaria were searched in PubMed, Scopus and Embase. The assessment of the quality of the included studies was conducted according to the Strengthening the Reporting of Observational Studies in Epidemiology guidelines. Qualitative and quantitative syntheses were performed to narratively describe and quantitatively pool the mean difference (MD) in TGF-β levels between uncomplicated malaria and healthy controls, and between severe and uncomplicated malaria, using a random-effects model. A total of 1027 relevant articles were identified, and 13 studies were included for syntheses. The meta-analysis results show 233 patients with uncomplicated malaria and 239 healthy controls. Patients with uncomplicated malaria (233 cases) had lower mean TGF-β levels than healthy controls (239 cases; *p* < 0.01, pooled MD = −14.72 pg/mL, 95% confidence interval (95% CI) = −20.46 to 8.99 pg/mL, I^2^ = 98.82%, seven studies). The meta-analysis found no difference in mean TGF-β levels between patients with severe malaria (367 cases) and patients with uncomplicated malaria (180 cases; *p* = 0.11, pooled MD = −6.07 pg/mL, 95% CI = −13.48 to 1.35 pg/mL, I^2^ = 97.73%, six studies). The meta-analysis demonstrated decreased TGF-β levels in patients with uncomplicated malaria compared to healthy controls. In addition, no difference in TGF-β levels was found between patients with severe and uncomplicated malaria. More research is needed to determine whether TGF-β levels could be a candidate marker for malarial infection or disease severity.

## 1. Introduction

Malaria is one of the world’s most serious public health issues. According to the 2021 World Malaria Report, nearly half of the world’s population lives in malaria-prone areas across 87 countries and territories [1]. In addition, malaria is expected to cause 241 million clinical episodes and 627,000 fatalities by 2020; the World Health Organization’s African Region is expected to account for 95% of all fatalities [1]. *Plasmodium falciparum* is the most common cause of malaria-related deaths, according to current evidence; however, *Plasmodium vivax*, *Plasmodium ovale* and *Plasmodium knowlesi* can also cause malaria-related deaths in a small number of cases [2,3,4,5].

The modulation of pro- and anti-inflammatory cytokines has been linked to various clinical presentations of malaria. During the acute phase of infection, a pro-inflammatory immune response suppresses parasite proliferation, with interferon-γ (IFN-γ) playing a key role [6]. Anti-inflammatory immune responses with interleukin (IL)-10 and transforming growth factor-β (TGF-β) play an important role in malaria pathophysiology [7]. TGF-β is a tissue-specific cytokine secreted by various cell types, including fibroblasts, epithelial cells, macrophages and other immune cells; it has several context-dependent immunomodulatory effects [8,9,10]. Controlling apoptosis, angiogenesis, wound healing, cancer growth and immunological modulation are all important functions of this protein [11,12,13,14,15]. TGF-β exists in three isoforms in mammals (TGF-β1, -β2 and -β3), with TGF-β1 being the most abundant isoform expressed in immune cells [16,17,18]. TGF-β regulates the development and activity of many immune cells. It promotes the differentiation of T helper 17 and 9, T follicular helper cells and regulatory T cells (Tregs), which function in immunosuppression [19]. In contrast, TGF-β signaling negatively affects the number of effector T cells that stimulate cellular and humoral immune responses. TGF-β signaling inhibits the development of Th1, T helper 2 and CD8^+^ T cells. TGF-β also inhibits B-cell growth, B cell survival and IgG class switching. It also inhibits the activity of natural killer cells [19].

TGF-β activity worsens several protozoal infections, including *Leishmania braziliensis* [20] and a helminth worm, *Heligmosomoides polygyrus* [21]. In contrast, TGF-β has been approved as a critical anti-inflammatory immunomodulator that aids in reducing inflammation and pathology during *Plasmodium* infection [9]. Previous research has shown that patients with acute malaria have higher TGF-β levels than the normal limit [22,23,24]. Lower TGF-β levels have previously been reported in patients with severe malaria [25,26,27]. Nevertheless, the role of TGF-β in acute malaria and the prevention of severe malaria are mostly unknown. Furthermore, studies on the role of TGF-β in various malarial types have relied on a small number of participants, which increased statistical error. As a result, this study used a meta-analysis approach to investigate the difference in TGF-β levels between several groups of malaria patients and healthy controls. This study would provide evidence-based information on TGF-β levels and malaria for future immunological research.

## 2. Methods

### 2.1. Protocol and Registration

The systematic review protocol was registered at PROSPERO (ID: CRD42022318864). The systematic review followed the Preferred Reporting Items for Systematic Reviews and Meta-Analyses checklist [28].

### 2.2. PICO Questions

The research questions were developed using the P (participants), I (intervention), C (comparator) and O (outcome) framework. P denotes malaria patients (severe or uncomplicated), I denotes not applicable (none), C denotes uncomplicated malaria or healthy controls and O denotes TGF-β levels in participants.

### 2.3. Eligibility Criteria

Inclusion criteria were studies that reported TGF-β levels in uncomplicated and healthy controls and TGF-β levels in severe and uncomplicated malaria patients. Eligible study designs included cross-sectional, cohort, case–control or observational studies. Exclusion criteria included in vivo and in vitro studies, TGF-β gene/protein expression, mosquito experiments, reviews, TGF-β in pregnancy/cord blood malaria, TGF-β in uncomplicated malaria only, TGF-β gene polymorphism, conference abstracts and TGF-β in co-infection. Cases where TGF-β data in malaria could not be extracted, and the presence of TGF-β only in severe malaria, full-text unavailability and systematic reviews were grounds for exclusion.

### 2.4. Information Sources

A comprehensive search was performed in three databases, including PubMed, Scopus and Embase, from 1 to 7 March 2022. The search terms (‘Milk Growth Factor’ OR ‘TGF-beta’ OR TGFbeta OR ‘Platelet Transforming Growth Factor’ OR ‘Bone-Derived Transforming Growth Factor’ OR ‘Bone Derived Transforming Growth Factor’ OR ‘transforming growth factor’) AND (‘malaria OR *Plasmodium*’) were used to search the potentially relevant studies. The details of the search strategy are found in Table S1. Searches of reference lists of the included studies and Google Scholar were also performed to ensure that the relevant studies were not missed during study selection.

### 2.5. Study Selection

Study selection was performed as follows: (i) duplicates that were identified from three databases were excluded; (ii) screening of titles and abstracts, and non-relevant studies were excluded, and (iii) the remaining studies were examined against the eligibility criteria and studies that did not meet the criteria were excluded for specific reasons. Studies were selected by two authors independently (MK and AM). Disagreements between authors during study selection were resolved by discussion and reaching a consensus.

### 2.6. Definitions

Severe malaria is characterised by the presence of malaria parasites in the blood of patients with one or more of the following complications: impaired consciousness, prostration, multiple convulsions, acidosis, hypoglycaemia, severe malarial anaemia, renal impairment, jaundice, pulmonary oedema, significant bleeding, shock and hyperparasitemia [29]. Uncomplicated malaria is characterised by the presence of malaria parasites in the blood of patients without the complications above. Healthy controls were participants residing in the same area who tested negative for malaria parasites and showed no signs or symptoms of malaria.

### 2.7. Data Extraction

Each study’s first author’s name, year of publication, study location, year of study conduction, TGF-β levels, *Plasmodium* spp., age groups, number of patients in each group, the method for malaria detection and method for TGF-β quantification were extracted. Data extraction was performed by two authors (MK and PK). Disagreements between authors during data extraction were resolved by discussion and reaching a consensus.

### 2.8. Quality of the Included Studies

The assessment of the quality of the included studies followed the Strengthening the Reporting of Observational Studies in Epidemiology statement [30]. Overall, 22 scores were given to judge the quality of each study. The quality of each study was categorised as high, moderate or low quality if they scored >75, 50 to 75 or <50 percentiles, respectively.

### 2.9. Outcomes

There were two outcomes in the meta-analysis: (1) pooled mean difference (MD) and 95% confidence interval (95% CI) of TGF-β levels between patients with uncomplicated malaria and healthy controls, and (2) pooled MD and 95% CI of TGF-β levels between patients who had severe malaria and uncomplicated malaria.

### 2.10. Data Syntheses

Data syntheses included qualitative and quantitative syntheses. To describe the difference in TGF-β levels across several groups of participants from all included studies that reported the same outcome, a qualitative synthesis was performed. Using a random-effects model, the quantitative synthesis was used to pool the MD of TGF-β levels (pg/mL) between uncomplicated malaria and healthy controls and between severe and uncomplicated malaria [31]. In the case of the median, rather than mean TGF-β levels reported in the included study, the mean and 95% CI were calculated using the median and interquartile range, as suggested previously [32]. The heterogeneity of the MD among the included studies was determined using Cochrane χ^2^ and I^2^ statistics for inconsistency. The Cochrane χ^2^ with *p* < 0.10 or an I^2^ of >25% indicated heterogeneity of MDs among the included studies. A meta-regression analysis was performed to determine the effect of covariates on the pooled MD. Subgroup analysis was further performed in the event of covariates confounding the pooled MD. Further sensitivity analysis using the leave-one-out method was undertaken to determine the robustness of the meta-analysis results. The publication bias was assessed using the funnel plot, Egger’s test and contour-enhanced funnel plot to determine the reporting bias across publications. All analyses, including forest plots, funnel plots and contour-enhanced funnel plots, were performed using Stata version 17.0 (StataCorp, College Station, TX, USA).

## 3. Results

### 3.1. Search Results

**Figure 1:**
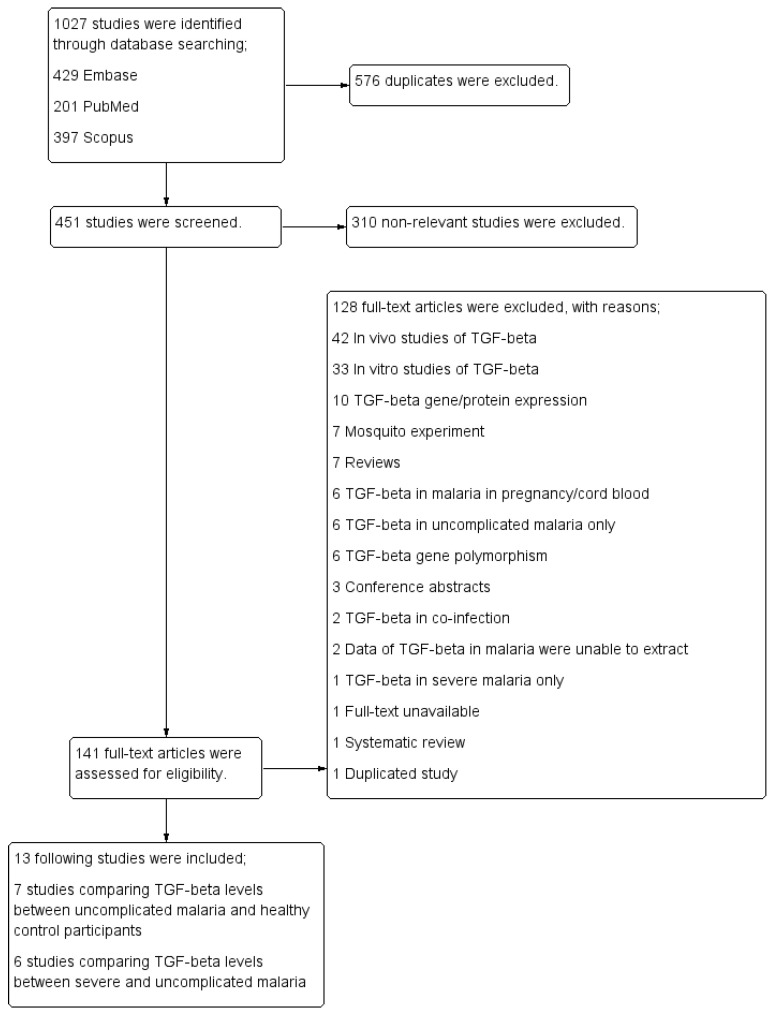
Study flow diagram.

A total of 1027 articles were retrieved from PubMed (201 articles), Scopus (397 articles) and Embase (429 articles). After 576 articles were excluded, 451 articles were screened for titles and abstracts. After 310 non-related articles were excluded, 141 articles were examined for full texts. For specific reasons, after the screening of full texts, the following articles were excluded: 42 were TGF-β in vivo studies, 33 were TGF-β in vitro studies, 10 were TGF-β gene/protein expression studies, 7 were mosquito experiments, 7 were reviews, 6 were TGF-β in pregnancy/cord blood, 6 were TGF-β in uncomplicated malaria only, 6 were TGF-β gene polymorphism studies, 3 were conference abstracts, 2 were TGF-β in co-infection, 1 was about TGF- β in severe malaria only, 1 had no full-text available, 1 was a systematic review, and 1 was a duplicate study. Finally, 13 studies were included [26,27,33,34,35,36,37,38,39,40,41,42,43]: 7 studies [33,34,35,36,37,38,39] comparing TGF-β levels between uncomplicated malaria and healthy controls and 6 studies [26,27,40,41,42,43] comparing TGF-β levels between severe and uncomplicated malaria (Figure 1).

### 3.2. Characteristics and Quality of the Included Studies

The characteristics of the included studies are shown in Table 1. The included studies were published between 1995 and 2021. These studies were prospective observational studies (six studies; 46.2%) [33,39,40,41,42,43], case–control studies (three studies; 23.1%) [27,35,37], cross-sectional studies (three studies; 23.1%) [34,36,38] and a cohort study (one study; 7.69%) [26]. The included studies were conducted in Africa (53.8%; Uganda [26,35,37], Gabon [33], Burkina Faso [40], Kenya and Uganda [41] and Gabon [42]), Asia (30.8%; Thailand [27,39], Indonesia [34], India [43]) and South America (15.4%; Brazil [36,38]). Most included studies (10 studies; 76.9%) enrolled patients with *P. falciparum* [26,27,33,35,37,39,40,41,42,43]. Six studies enrolled children (46.2%) [26,33,34,40,41,42], four enrolled adults (30.8%) [36,37,38,39] and three studies enrolled all age groups (23.1%) [27,34,43]. Thirteen studies enrolled 367 patients with severe malaria, 362 uncomplicated malaria and 358 healthy controls. Most included studies (9 studies; 69.2%) [26,27,33,35,38,39,40,42,43] used microscopic examination for the detection of malaria parasites and used enzyme-linked immunosorbent assays (ELISA) for the quantification of TGF-β levels (11 studies; 84.6%) [26,27,33,34,35,37,39,40,41,42,43]. Details of the included studies are shown in Table S2. All 13 studies included in the systematic review were of high quality (Table S3). No study was excluded from the meta-analysis. 

### 3.3. TGF-β Levels between Uncomplicated Malaria and Healthy Controls

Among the seven studies that enrolled patients with severe and uncomplicated malaria [33,34,35,36,37,38,39], six studies demonstrated lower mean TGF-β levels in patients with uncomplicated malaria than in healthy controls [26,27,33,38,39,42]. A study in Burkina Faso [40] demonstrated no difference in mean TGF-β levels between the two groups. Another study in Indonesia [34] demonstrated that TGF-β levels were higher in *P. falciparum* monoinfection and mixed *P. falciparum*/*P. vivax* infections than those in healthy controls; there was no difference in TGF-β levels between patients with *P. vivax* monoinfection and healthy controls. Two studies in Uganda [35,37] demonstrated that patients with malaria had higher median TGF-β levels than healthy controls. Another study in Brazil [35] demonstrated patients with uncomplicated malaria had higher TGF-β levels than healthy controls, but the median TGF-β levels were similar to endemic controls.

**Figure 2:**
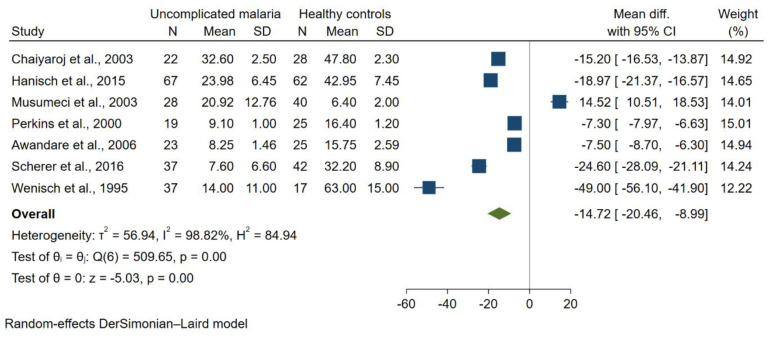
Funnel plot demonstrating a difference in mean TGF-β levels (pg/mL) in uncomplicated malaria and healthy controls [26,27,33,38,39,40,42]. Grey *y*-axis line, line of no effect size; blue square boxes, mean TGF-β levels from each study; line on the left and right of blue square boxes, 95% CI; green diamond; overall effect size; I^2^, H^2^ and τb^2^, heterogeneity measures; test of θ, overall effect size. Abbreviation: SD, standard deviation.

The difference in TGF-β levels between patients with uncomplicated malaria and healthy controls was estimated using available data from seven studies [26,27,33,38,39,40,42] that enrolled 233 patients with uncomplicated malaria and 239 healthy controls. The meta-analysis showed lower mean TGF-β levels in patients with uncomplicated malaria than healthy controls (*p* < 0.01, pooled MD = −14.72 pg/mL, 95% CI = −20.46 to 8.99 pg/mL, I^2^ = 98.82%; seven studies; Figure 2). The meta-regression analysis using study design, continent, age group, *Plasmodium* spp., method for malaria detection and method for TGF-β quantification as covariates demonstrated that continent and age group confounded the pooled MD (*p* < 0.01 from each analysis); therefore, subgroup analyses of continent and age group were further performed.

**Figure 3:**
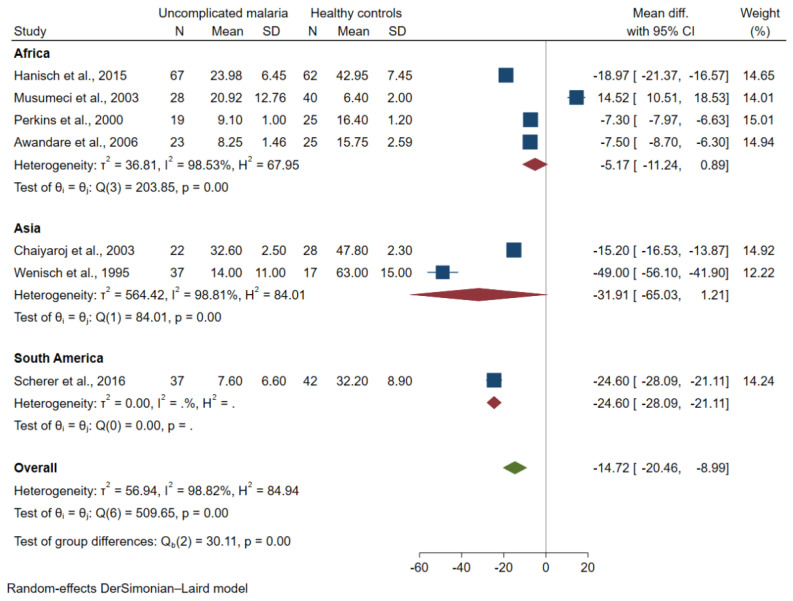
Forest plot demonstrating a difference in mean TGF-β levels (pg/mL) in uncomplicated malaria and healthy controls by continents [26,27,33,38,39,40,42]. Grey *y*-axis line, line of no effect size; blue square boxes, mean TGF-β levels from each study; line on the left and right of blue square boxes, 95% CI; green diamond; overall effect size; crimson diamond, overall effect size in each subgroup; I^2^, H^2^ and τb^2^, heterogeneity measures.

**Figure 4:**
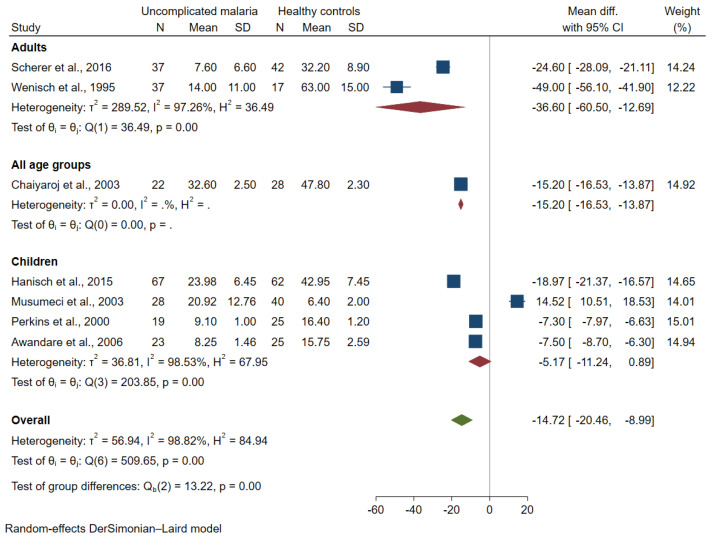
Forest plot demonstrating a difference in mean TGF-β levels (pg/mL) in uncomplicated malaria and healthy controls by age groups [26,27,33,38,39,40,42]. Grey *y*-axis line, line of no effect size; blue square boxes, mean TGF-β levels from each study; line on the left and right of blue square boxes, 95% CI; green diamond; overall effect size; crimson diamond, overall effect size in each subgroup; I^2^, H^2^ and τb^2^, heterogeneity measures.

The subgroup analysis of continent demonstrated that no difference in mean TGF-β levels between the two groups was found among studies in Africa (pooled MD = −5.17 pg/mL, 95% CI = −11.24 to 0.89 pg/mL, I^2^ = 98.53%; four studies) and Asia (pooled MD = −31.91 pg/mL, 95% CI = −65.03 to 1.21 pg/mL, I^2^ = 98.81%; two studies; Figure 3). The subgroup analysis of age group demonstrated lower mean TGF-β levels in patients with uncomplicated malaria than healthy controls among studies that enrolled adults (pooled MD = −36.6 pg/mL, 95% CI = −60.5 to −12.69 pg/mL, I^2^ = 97.26%; two studies). No difference in mean TGF-β levels between the two groups was found among studies that enrolled children (pooled MD = −5.17 pg/mL, 95% CI = −11.24 to 0.89 pg/mL, I^2^ = 98.53%; four studies; Figure 4).

The difference in TGF-β levels between children with uncomplicated *P. falciparum* malaria and healthy controls was estimated using available data from four studies [26,33,40,42]. The meta-analysis demonstrated no difference in mean TGF-β levels between the two groups (*p* = 0.09, pooled MD = −5.17 pg/mL, 95% CI = −11.24 to 0.89 pg/mL, I^2^ = 98.53%; four studies; Supplementary Figure S1). The difference in TGF-β levels between adults with uncomplicated *P. vivax* malaria and healthy controls was estimated using available data from three studies [27,33,39]. The meta-analysis demonstrated lower mean TGF-β levels in adults with uncomplicated *P. vivax* malaria than healthy controls (*p* < 0.01, pooled MD = −22.57 pg/mL, 95% CI = −32.38 to −12.75 pg/mL, I^2^ = 98.88%; three studies; Supplementary Figure S2).

### 3.4. TGF-β Levels between Severe and Uncomplicated Malaria

Among the six studies that enrolled patients with severe and uncomplicated malaria [26,27,40,41,42,43], three studies demonstrated lower mean TGF-β levels in patients with severe malaria than those with uncomplicated malaria [26,27,42]. Two studies demonstrated no difference in mean TGF-β levels between the two groups [40,41]. A study in India demonstrated higher median TGF-β levels in patients with severe malaria than those with uncomplicated malaria [43].

**Figure 5:**
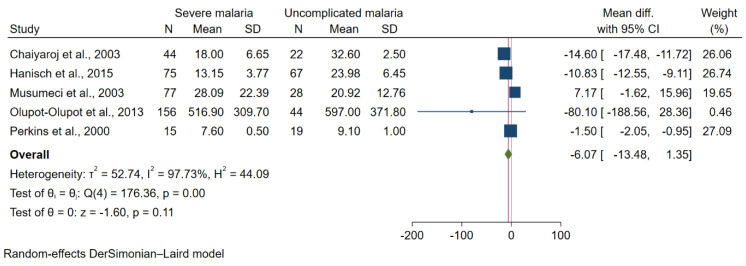
Forest plot demonstrating a difference in mean TGF-β levels (pg/mL) in severe and uncomplicated malaria [26,27,40,41,42]. Grey *y*-axis line, line of no effect size; blue square boxes, mean TGF-β levels from each study; line on the left and right of blue square boxes, 95% CI; green diamond; overall effect size; I^2^, H^2^ and τb^2^, heterogeneity measures; test of θ, overall effect size.

The difference in TGF-β levels between severe and uncomplicated malaria was estimated using available data from six studies that enrolled 367 patients with severe malaria and 180 patients with uncomplicated malaria [26,27,40,41,42]. The meta-analysis results show no difference in mean TGF-β levels between the two groups (*p* = 0.11, pooled MD = −6.07 pg/mL, 95% CI = −13.48 to 1.35 pg/mL, I^2^ = 97.73%; five studies; Figure 5). The meta-regression analysis using study design, continent, age group, *Plasmodium* spp., method for malaria detection, and method for TGF-β quantification as covariates demonstrated that these covariates did not confound the pooled MD (*p* > 0.05 from each analysis); therefore, subgroup analysis was not further performed. The difference in TGF-β levels between children with severe and uncomplicated *P. falciparum* malaria was estimated using available data from four studies [26,40,41,42]. The meta-analysis results show no difference in mean TGF-β levels between the two groups (*p* = 0.44, pooled MD = −3.12 pg/mL, 95% CI = −11.01 to 4.76 pg/mL, I^2^ = 97.26%; four studies; Supplementary Figure S3).

### 3.5. Sensitivity Analysis

**Figure 6:**
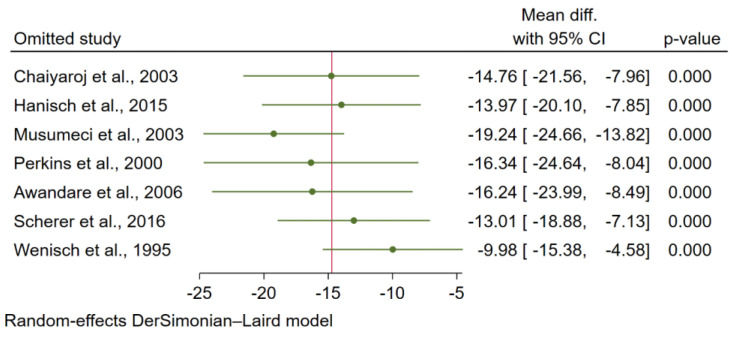
Sensitivity analysis determining the robustness of the meta-analysis results of the difference in TGF-β levels (pg/mL) between uncomplicated malaria and healthy controls [26,27,33,38,39,40,42].

**Figure 7:**
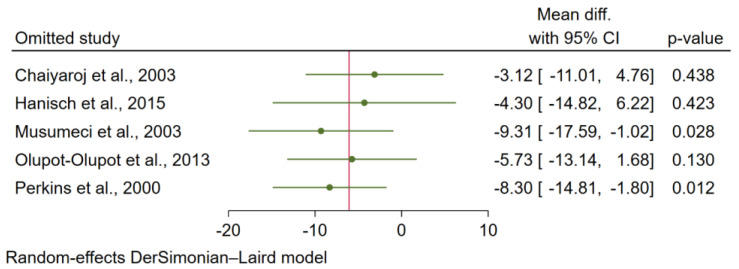
Sensitivity analysis determining the robustness of the meta-analysis results of the difference in TGF-β levels (pg/mL) between severe and uncomplicated malaria [26,27,40,41,42].

Sensitivity analysis was performed to determine the robustness of the meta-analysis results. When each study was excluded from the meta-analysis of the difference in TGF-β levels between patients with uncomplicated malaria and healthy controls, the re-run analysis showed robust meta-analysis results (*p* < 0.05 in each analysis; Figure 6). In addition, when each study was excluded from the meta-analysis of the difference in TGF-β levels between patients with severe and uncomplicated malaria, the re-run analysis showed that the meta-analysis results were not robust (*p* < 0.05 in two analyses and *p* > 0.05 in three analyses; Figure 7).

### 3.6. Publication Bias

**Figure 8:**
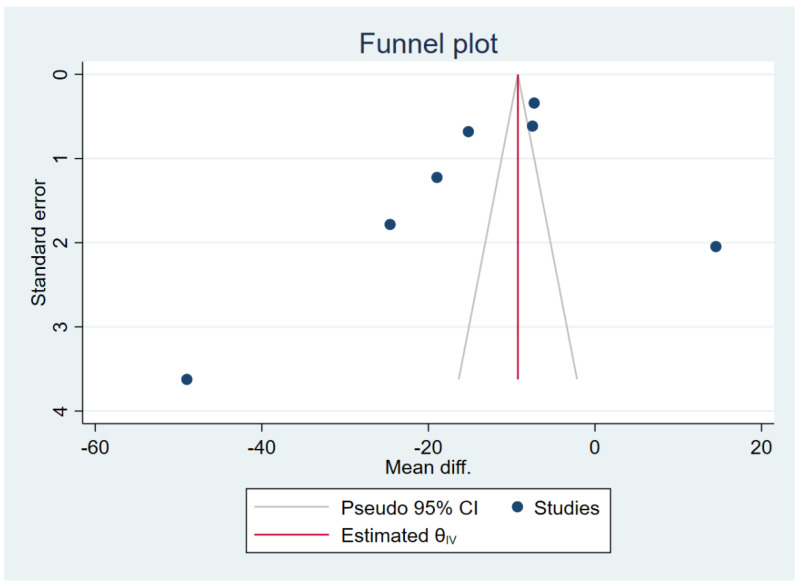
Funnel plot demonstrating the MD in TGF-β levels between uncomplicated malaria and healthy controls (pg/mL). The plot shows an asymmetrical distribution of the effect estimates (blue dots) from the middle line (red line). Abbreviations: Mean diff., MD; Estimated θ_IV_, overall effect size.

**Figure 9:**
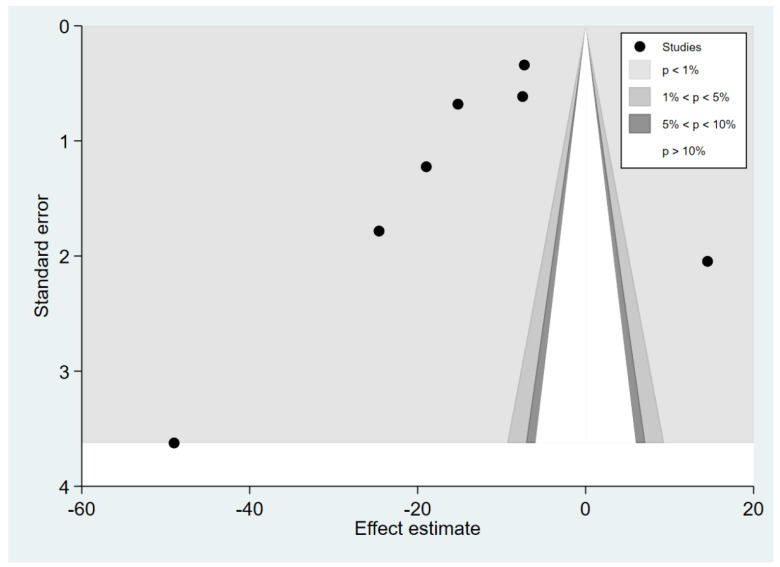
Contour-enhanced funnel plot demonstrating the MD in TGF-β levels between uncomplicated malaria and healthy controls (pg/mL) in the significant area (pale grey area), indicating that the cause of funnel plot asymmetry was the publication bias.

The publication bias was assessed by the visualisation of the funnel plot, Egger’s test and contour-enhanced funnel plot. The meta-analysis results of the difference in TGF-β levels between patients with uncomplicated malaria and healthy controls reveal funnel plot asymmetry (Figure 8). The Egger’s test demonstrated a small-study effect (*p* = 0.002). The contour-enhanced funnel plot revealed that MDs were only distributed in a significant area (*p* = 0.01; Figure 9), indicating that the meta-analysis of the difference in TGF-β levels between patients with uncomplicated malaria and healthy controls had a publication bias. The trim and fill method has been applied to correct the pooled MD in the presence of a publication bias. After the publication was adjusted, the meta-analysis results showed that patients with uncomplicated malaria had lower mean TGF-β levels than healthy controls (pooled MD = −9.25 pg/mL, 95% CI = −9.76 to 8.74 pg/mL; 13 studies).

**Figure 10:**
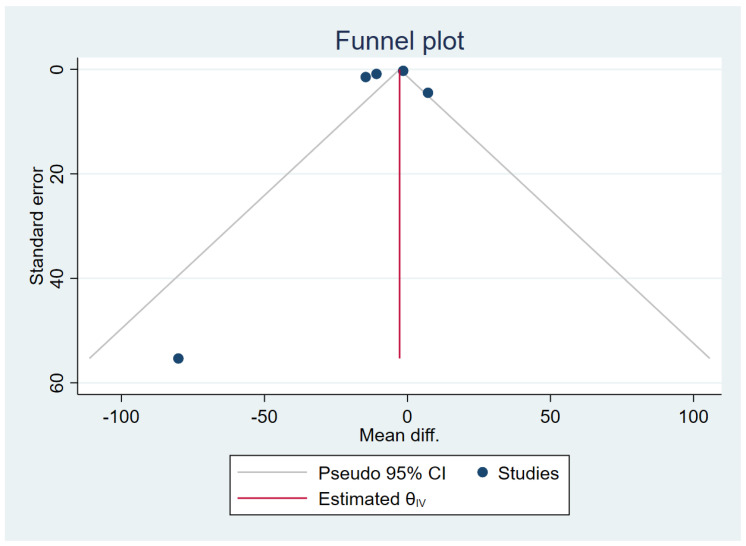
Funnel plot demonstrating the MD in TGF-β levels between severe and uncomplicated malaria (pg/mL). The plot shows an asymmetrical distribution of the effect estimates (blue dots) from the middle line (red line).

**Figure 11:**
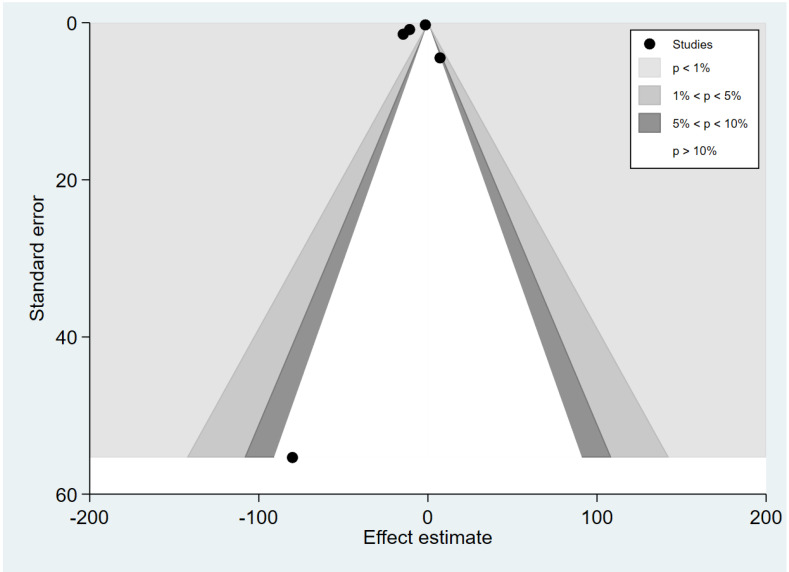
Contour-enhanced funnel plot demonstrating the MD in TGF-β levels between severe and uncomplicated malaria (pg/mL) in the significant area (pale grey area) and non-significant areas (white and dark grey colours), indicating that the cause of funnel plot asymmetry was the heterogeneity of the effect estimates and the publication bias.

The meta-analysis results of the difference in TGF-β levels between patients with severe and uncomplicated malaria reveal funnel plot asymmetry (Figure 10). The Egger’s test demonstrated no small-study effect (*p* = 0.45). The contour-enhanced funnel plot revealed that the MDs were distributed in both significant (*p* = 0.01) and non-significant (*p* > 0.05, Figure 11) areas, implying that the meta-analysis of the difference in TGF-β levels between patients with severe and uncomplicated malaria may have been influenced by the publication bias. The trim and fill method has been applied to correct the pooled MD in the presence of the publication bias. After the publication was adjusted, the meta-analysis results show that patients with severe malaria had lower mean TGF-β levels than those with uncomplicated malaria (pooled MD = −2.75 pg/mL, 95% CI = −3.27 to −2.23 pg/mL; 13 studies).

## 4. Discussion

In this study, the meta-analysis results show that TGF-β levels were significantly lower in malaria patients than in healthy controls, suggesting that lower TGF-β levels might be a candidate marker for acute malaria. The certainty of the evidence was confirmed by the sensitivity analysis. Only Musumeci et al. [40] found higher TGF-β levels than healthy controls, implying that TGF-β has two different functions in malarial infection depending on the stage of infection. TGF-β stimulates Th1-mediated pathways that limit parasite development early in the infection. In contrast, TGF-β suppresses the Th1 response, limiting inflammation-related diseases [40].

In a previous study, despite increased parasitemia levels, high TGF-β levels were associated with lower clinical states of malaria [9]. TGF-β was elevated in malaria co-infection, and might be used to differentiate malaria monoinfection from malaria co-infections [35,37]. Nevertheless, TGF-β levels were lower in patients with uncomplicated malaria than in healthy controls in subgroup analyses of adults enrolled in studies [38,39]. No difference in mean TGF-β levels was found between the two groups in studies that included children [26,33,40,42]. Comparing similar age groups and *Plasmodium* spp., no difference in TGF-β levels in children with uncomplicated *P. falciparum* malaria and healthy controls was found based on the meta-analysis results. The trend for TGF-β levels in children presenting with *P. falciparum* malaria during the acute phase of the infection was lower than in healthy controls, because 75% of the studies included in the meta-analysis demonstrated lower TGF-β levels in children. Nevertheless, the high heterogeneity of the meta-analysis results raises the question of whether higher or lower TGF-β levels could be a candidate marker for *P. falciparum* malaria in children. In healthy children 0–14 years old, there were significantly higher serum TGF-β levels than adults >15 years old [44]. It is possible that, after acute malarial infection in children, regulatory and pro-inflammatory cytokine responses might be suppressed in younger children; compared to healthy children, there might be lower serum TGF-β levels in children infected with malaria, as shown by the meta-analysis results. For *P. vivax* infection in adults, the meta-analysis results show significantly lower TGF-β levels in adults with *P. vivax* malaria than healthy controls. There was a strong trend towards TGF-β levels in adults presenting with *P. vivax* malaria during the acute phase of the infection, as all studies included in the meta-analysis demonstrated lower TGF-β levels in children [27,33,39]. Therefore, lower TGF-β levels might be a candidate marker for *P. vivax* malaria in adults. There is a possible explanation for the lower TGF-β levels among adults infected with malaria. TGF-β levels might be suppressed by repeated malarial exposure, age-related variations in the immune response, or both [45]. Nonetheless, a comparison of TGF-β levels in children from a low transmission area versus a high transmission area revealed a significant difference in TGF-β levels, with children from the low transmission area having higher TGF-β levels [23]. As a result, differences in malaria endemicity could be another source of TGF-β heterogeneity among the included studies. This continent subgroup analysis revealed no subgroup differences in TGF-β levels between studies in Africa and Asia, indicating a need for additional research.

The meta-analysis results confirm no difference in mean TGF-β levels between severe and uncomplicated malaria. Considering the same age groups and *Plasmodium* spp., no difference in TGF-β levels in children with severe and uncomplicated *P. falciparum* malaria was found based on the meta-analysis results. With the high heterogeneity of TGF-β levels in the included studies, it raises the question of whether higher or lower TGF-β levels could be a candidate marker for severe *P. falciparum* malaria in children. According to Musumeci et al. [40], higher TGF-β levels downregulate IL-12, modulating the immune response to *P. falciparum* and thus decreasing susceptibility to severe malaria. Chaiyaroj et al. [27] found that TGF-β levels were significantly lower in all malaria groups compared to controls, with the lowest levels of TGF-β detected in patients with cerebral malaria. Hanisch et al. showed that decreased TGF-β was associated with cytokine/chemokine changes associated with disease severity and death; however, no link between parasitemia and TGF-β was discovered [26]. TGF-β was inversely related to infection severity in the *Plasmodium* *berghei*-infected mouse model, and is critical for controlling cytokine expression by significantly lowering TGF-β plasma levels [46]. Furthermore, the low TGF-β/tumour necrosis factor-α (TNF-α) ratio implies that a lack of TGF-β response in children with severe malaria may result in TNF-α overproduction [43]. In patients with severe malaria, TNF-α production was higher than those with uncomplicated malaria [47]. TGF-β administration prevents mortality during lethal infections in mice by neutralising the IFN-γ pathway and downregulating TNF-α production [48]. TGF-β levels were also highest in cerebral malaria, followed by severe malaria, then non-severe malaria [43]. Increased TGF-β production, the presence of CD4^+^CD25^+^FOXP3^+^ Tregs, and a reduction in pro-inflammatory cytokine production have all been linked to higher parasite development rates in *P. falciparum*-infected volunteers [49]. TGF-β levels may be comparable in patients with severe and uncomplicated malaria because this cytokine may follow a pattern dictated by other factors rather than directly influencing the pathogenic process.

This study has certain limitations. First, although the meta-regression revealed that patient age confounds the pooled effect measure between patients with uncomplicated malaria and healthy controls, heterogeneity within each subgroup remained considerable. Other factors that could alter the pooled effect measure were discovered as a result of these findings, and they were examined further. Second, there are few studies on TGF-β levels in severe and uncomplicated malaria. As a result, the meta-analysis results revealed a diverse set of results with publication bias. If more papers were included in the meta-analysis, TGF-β levels might be lower in patients with severe malaria than in those with uncomplicated malaria, according to publication bias adjustment.

## 5. Conclusions

This meta-analysis showed decreased TGF-β levels in patients with uncomplicated malaria compared to healthy controls. There was no difference in TGF-β levels in children with uncomplicated *P. falciparum* malaria compared to healthy controls, but a significant decrease in TGF-β levels was observed among adults with uncomplicated *P. vivax* malaria compared to healthy controls. In addition, there was no difference in TGF-β levels between patients with severe and uncomplicated malaria. More research is needed to determine the TGF-β levels as a candidate marker for malarial infection and severity.

## Figures and Tables

**Table 1:** Characteristics of the included studies.

Characteristics	n	%
Study designs		
Prospective observational studies	6	46.2
Case–control studies	3	23.1
Cross-sectional studies	3	23.1
Cohort study	1	7.69
Study areas		
Africa	7	53.8
Asia	4	30.8
South America	2	15.4
*Plasmodium* spp.		
*P*. *falciparum*	10	76.9
*P*. *vivax*	2	15.4
*P*. *falciparum*/*P*. *vivax*/mixed infection	1	7.69
Participants		
Children	6	46.2
Adults	4	30.8
All age groups	3	23.1
Methods for malaria detection		
Microscopy	9	69.2
Microscopy/RDT	2	15.4
Microscopy/PCR	2	15.4
Methods for TGF-β quantification		
ELISA	11	84.6
Bead-based assay	2	15.4

Abbreviations: PCR, polymerase chain reaction; RDT, rapid diagnostic tests.

## Data Availability

All data related to the manuscript were available in the main manuscript its supplementary files.

## Supplementary Materials

The following supporting information can be downloaded at: https://www.mdpi.com/article/10.3390/tropicalmed7100299/s1, Figure S1: Uncomplicated Pf children vs controls; Figure S2: Uncomplicated Pv adults vs controls; Figure S3: Severe vs uncomplicated Pf children; Table S1: Search term; Table S2: Details of the included studies; Table S3: Quality of the included studies.
