# Supplementary material for: Transforming Growth Factor-β Concerning Malarial Infection and Severity: A Systematic Review and Meta-Analysis
Source: Trop Med Infect Dis. 2022 Oct 13;7(10):299. doi: 10.3390/tropicalmed7100299 (PMC9612234; doi:10.3390/tropicalmed7100299)
Supplement: Supplementary file 1 [file tropicalmed-07-00299-s001.zip › Table S1. Search term.pdf]

Tropical Medicine and Infectious Disease

MDPI

Systematic Review

Transforming Growth Factor-β Concerning Malarial Infection and Severity: A Systematic Review and Meta-Analysis

Kwuntida Uthaisar Kotepui [1,†], Pattamaporn Kwankaew [1,2†], Frederick Ramirez Masangkay [3], Aongart Mahittikorn [4,*] and Manas Kotepui [1,*]

[1] Medical Technology, School of Allied Health Sciences, Walailak University, Tha Sala 80160, Thailand;
[4] Research Excellence Center for Innovation and Health Product, Walailak University, Tha Sala 80161, Thailand
[2] Department of Medical Technology, Faculty of Pharmacy, University of Santo Tomas, Manila 1008, Philippines;
[3] Department of Protozoology, Faculty of Tropical Medicine, Mahidol University, Bangkok 10400, Thailand
* Correspondence: aongart.mah@mahidol.ac.th (A.M.); manas.ko@wu.ac.th (M.K.)
† These authors contributed equally to this work.

Table S1. Search term.

Databases	Search terms/Search strategy	Date
MEDLINE (via PubMed)	("Milk Growth Factor" OR "TGF-beta" OR TGFbeta OR "Platelet Transforming Growth Factor" OR "Bone-Derived Transforming Growth Factor" OR "Bone Derived Transforming Growth Factor" OR "transforming growth factor") AND (malaria OR plasmodium)<br>Search results: 201	1 to 7 March 2022
Scopus	("Milk Growth Factor" OR "TGF-beta" OR TGFbeta OR "Platelet Transforming Growth Factor" OR "Bone-Derived Transforming Growth Factor" OR "Bone Derived Transforming Growth Factor" OR "transforming growth factor") AND (malaria OR plasmodium)<br>Search option: Title, abstract, keywords<br>Search results: 397	1 to 7 March 2022
Embase	("Milk Growth Factor" OR "TGF-beta" OR TGFbeta OR "Platelet Transforming Growth Factor" OR "Bone-Derived Transforming Growth Factor" OR "Bone Derived Transforming Growth Factor" OR "transforming growth factor") AND (malaria OR plasmodium)<br>Search option: All fields<br>Search results: 429	1 to 7 March 2022
