# Supplementary material for: Transforming Growth Factor-β Concerning Malarial Infection and Severity: A Systematic Review and Meta-Analysis
Source: Trop Med Infect Dis. 2022 Oct 13;7(10):299. doi: 10.3390/tropicalmed7100299 (PMC9612234; doi:10.3390/tropicalmed7100299)
Supplement: Supplementary file 1 [file tropicalmed-07-00299-s001.zip › Table S3. Quality the included studies.pdf]

Systematic Review

Transforming Growth Factor-β Concerning Malarial Infection and Severity: A Systematic Review and Meta-Analysis

Kwuntida Uthaisar Kotepui [1,†], Pattamaporn Kwankaew [1,2†], Frederick Ramirez Masangkay [3], Aongart Mahittikorn [4,*] and Manas Kotepui [1,*]

[1] Medical Technology, School of Allied Health Sciences, Walailak University, Tha Sala 80160, Thailand;
[4] Research Excellence Center for Innovation and Health Product, Walailak University, The Sala, 80161, Thailand
[2] Department of Medical Technology, Faculty of Pharmacy, University of Santo Tomas, Manila 1008, Philippines;
[3] Department of Protozoology, Faculty of Tropical Medicine, Mahidol University, Bangkok 10400, Thailand
* Correspondence: aongart.mah@mahidol.ac.th (A.M.); manas.ko@wu.ac.th (M.K.)
† These authors contributed equally to this work.

Table S3. Quality of the included studies.

	Study	Score (out of 22)	Score (percentage)	Quality
Case-control studies	Bwanika et al., 2018	20	91	High
	Chaiyaroj et al., 2003	18	82	High
	Nsubuga et al., 2019	21	96	High
Cross-sectional studies	Hojo-Souza et al., 2017	18	82	High
Prospective observational studies	Awandare et al., 2006	21	96	High
	Hanisch et al., 2015	20	91	High
	Musumeci et al., 2003	18	82	High
	Olupot-Olupot et al., 2013	20	91	High
	Perkins et al., 2000	19	86	High
	Prakash et al., 2006	19	86	High
	Wenisch et al., 1995	18	82	High

STROBE: Strengthening the Reporting of Observational Studies in Epidemiology.
