# Supplementary figures and images for: Transforming Growth Factor-β Concerning Malarial Infection and Severity: A Systematic Review and Meta-Analysis
Source: Trop Med Infect Dis. 2022 Oct 13;7(10):299. doi: 10.3390/tropicalmed7100299 (PMC9612234; doi:10.3390/tropicalmed7100299)

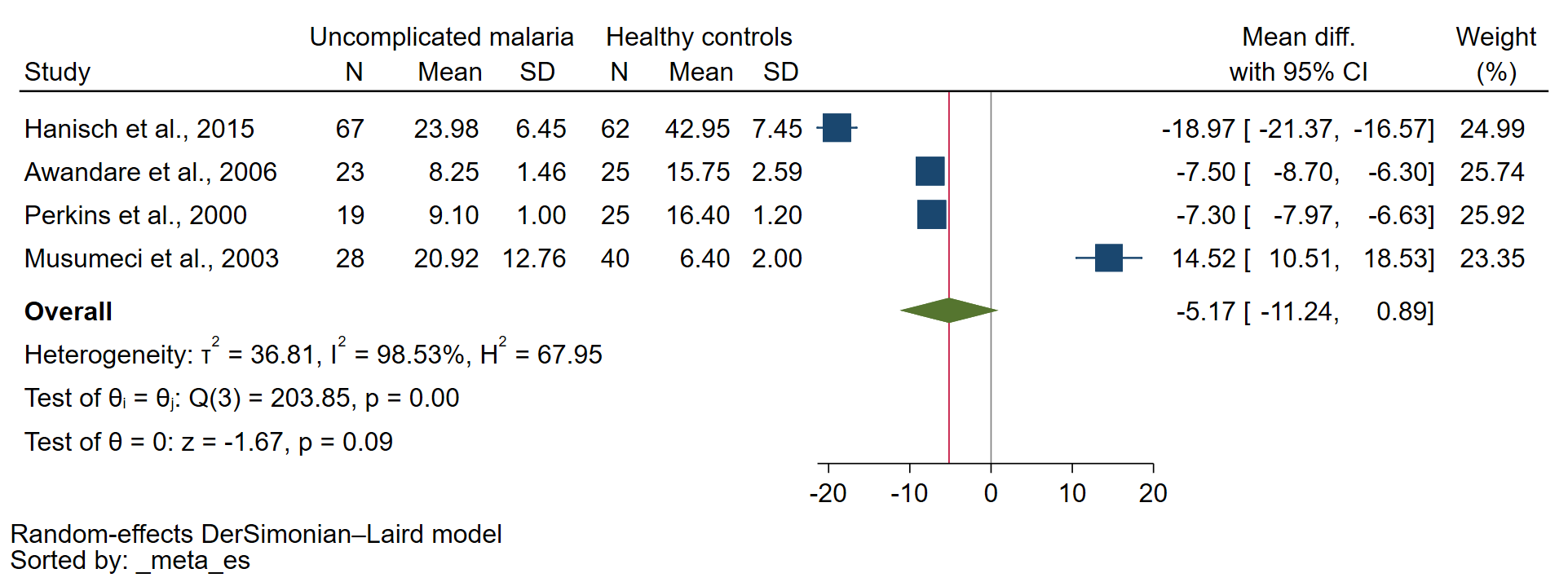

Supplement: Supplementary file 1 [file tropicalmed-07-00299-s001.zip › Supplementary Figure S1. Uncomplicated_Pf_children vs controls.tif]

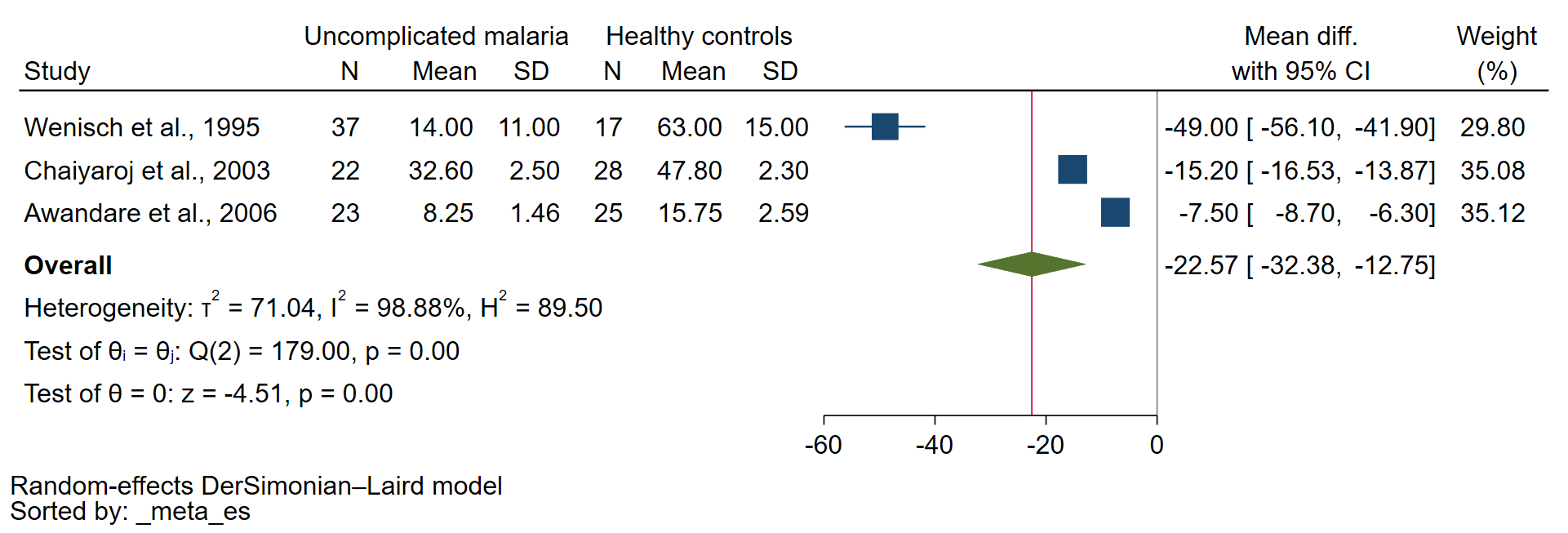

Supplement: Supplementary file 1 [file tropicalmed-07-00299-s001.zip › Supplementary Figure S2. Uncomplicated_Pv_adults vs controls.tif]

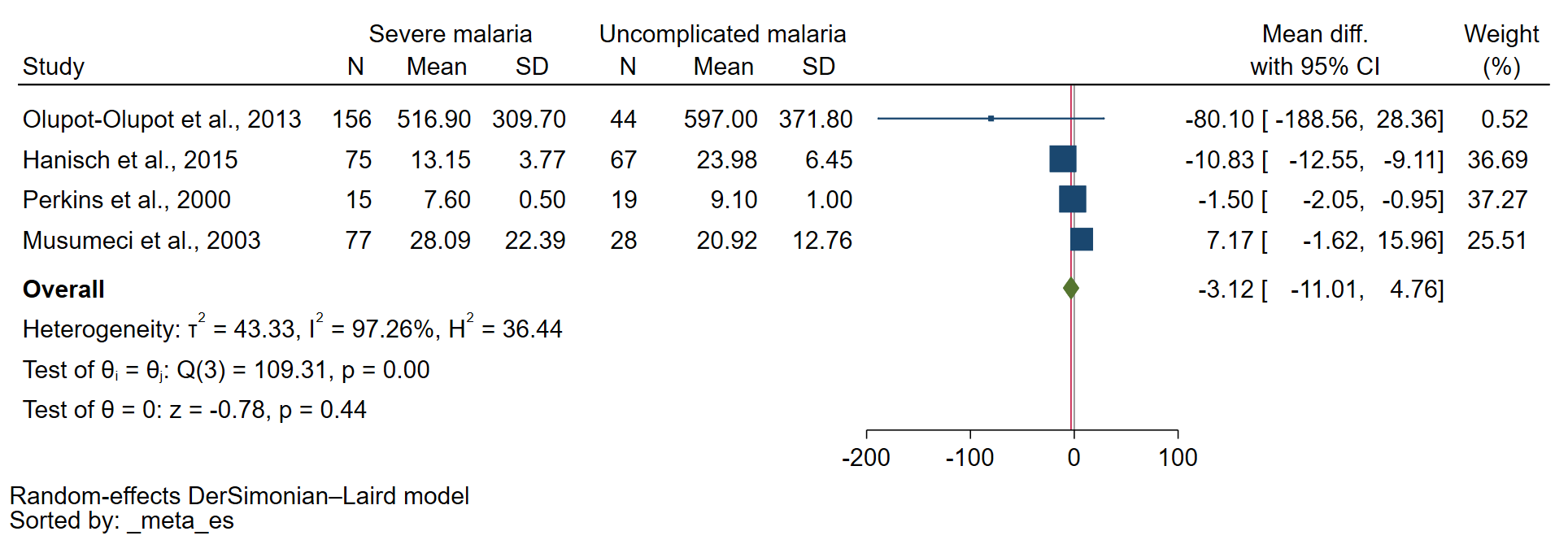

Supplement: Supplementary file 1 [file tropicalmed-07-00299-s001.zip › Supplementary Figure S3. Severe vs uncomplicated_Pf_children.tif]
